# Health*e*Steps™ Study Protocol: a pragmatic randomized controlled trial promoting active living and healthy lifestyles in at-risk Canadian adults delivered in primary care and community-based clinics

**DOI:** 10.1186/s12889-017-4047-8

**Published:** 2017-02-07

**Authors:** Dawn P. Gill, Wendy Blunt, Cassandra Bartol, Roseanne W. Pulford, Ashleigh De Cruz, P. Karen Simmavong, Adam Gavarkovs, Ian Newhouse, Erin Pearson, Bayley Ostenfeldt, Barbi Law, Kristina Karvinen, Pertice Moffit, Gareth Jones, Cori Watson, Guangyong Zou, Robert J. Petrella

**Affiliations:** 1 Centre for Studies in Family Medicine, Department of Family Medicine, Western Centre for Public Health and Family Medicine, 2nd Floor, Schulich School of Medicine and Dentistry, Western University, 1465 Richmond St., London, ON N6G 2M1 Canada; 2 Lawson Health Research Institute, London, Ontario Canada; 3 School of Health Studies, Faculty of Health Sciences, Western University, London, Ontario Canada; 4 School of Public Health, Brown University, Providence, Road Island USA; 5 School of Kinesiology, Lakehead University, Thunder Bay, Ontario Canada; 6 Centre for Education and Research on Aging and Health, Lakehead University, Thunder Bay, Ontario Canada; 7 School of Physical and Health Education, Nipissing University, North Bay, Ontario Canada; 8 Aurora Research Institute, Aurora College, Yellowknife, Northwest Territories Canada; 9 School of Health and Exercise Sciences, University of British Columbia, Kelowna, British Columbia Canada; 10 Northwest Local Health Integration Network, Chronic Disease, Health and Design Development, Thunder Bay, Ontario Canada; 11 Department of Epidemiology and Biostatistics, Schulich School of Medicine and Dentistry, Western University, London, Ontario Canada; 12 Robarts Clinical Trials, Robarts Research Institute, Western University, London, Ontario Canada; 13 School of Kinesiology, Faculty of Health Sciences, Western University, London, Ontario Canada

**Keywords:** Physical activity, Sedentary behaviour, Healthy eating, Primary care intervention, Chronic disease prevention and management, Health technology, Behaviour change, Lifestyle coaching

## Abstract

**Background:**

Physical inactivity is one of the leading causes of chronic disease in Canadian adults. With less than 50% of Canadian adults reaching the recommended amount of daily physical activity, there is an urgent need for effective programs targeting this risk factor. Health*e*Steps™ is a healthy lifestyle prescription program, developed from an extensive research base to address risk factors for chronic disease such as physical inactivity, sedentary behaviour and poor eating habits. Health*e*Steps™ participants are provided with in-person lifestyle coaching and access to *e*Health technologies delivered in community-based primary care clinics and health care organizations.

**Method/Design:**

To determine the effectiveness of Health*e*steps™, we will conduct a 6-month pragmatic randomized controlled trial with integrated process and economic evaluations of Health*e*Steps™ in 5 clinic settings in Southwestern Ontario. 110 participants will be individually randomized (1:1; stratified by site) to either the intervention (Health*e*Steps™ program) or comparator (Wait-list control). There are 3 phases of the Health*e*Steps™ program, lasting 6 months each. The *active phase* consists of bi-monthly in-person coaching with access to a full suite of *e*Health technology supports. During the *maintenance phase I*, the in-person coaching will be removed, but participants will still have access to the full suite of *e*Health technology supports. In the final stage, *maintenance phase II*, access to the full suite of *e*Health technology supports is removed and participants only have access to publicly available resources and tools.

**Discussion:**

This trial aims to determine the effectiveness of the program in increasing physical activity levels and improving other health behaviours and indicators, the acceptability of the Health*e*Steps™ program, and the direct cost for each person participating in the program as well as the costs associated with delivering the program at the different community sites. These results will inform future optimization and scaling up of the program into additional community-based primary care sites.

**Trial registration:**

NCT02413385 (Clinicaltrials.gov). Date Registered: April 6, 2015.

## Background

Physical activity (PA) is one of the most modifiable risk factors for preventing chronic disease, yet less than 50% of Canadian adults are considered physically active [1, 2] and only a third of adults meet the recommendation of 10,000 steps per day [3]. The high level of physical inactivity combined with a poor diet amongst adults has led to higher rates of chronic disease, such as obesity, type 2 diabetes, cardiovascular disease, hypertension, cancer, osteoporosis, depression, and premature death [1–6]. Furthermore, the cost of physical inactivity in Canada is estimated at $6.8 billion per year, representing approximately 3.7% of all direct and indirect government spending [1, 7]. Fortunately, modest increases in levels of PA can reduce this spending by $2.6 billion over the next 25 years, making low-cost and effective healthy lifestyle interventions that target physical activity an important public health and economic priority in Canada [2, 7–9]. Living a physically active lifestyle provides numerous health benefits, including protection from chronic diseases and improved quality of life [1]. In fact, being physically active is associated with a greater than 50% reduction in risk for premature death, and even small improvements in physical fitness can significantly reduce one’s risk for chronic disease [1].

Research shows that many chronic diseases can be prevented by decreasing four main behavioural risk factors: physical inactivity, poor nutrition, smoking and alcohol consumption [5, 10]. Public health organizations have worked to develop many resources to address these risk factors; however, previous research has noted that those who are the most physically inactive are not aware of these resources [11] or may be unsure of how to use this information to make a change. While research interventions promoting PA and healthy eating in controlled settings have shown unequivocal results in reducing chronic disease risk under certain conditions, effective methods for implementing this evidence into everyday primary care practice requires further exploration [12–15]. This is particularly true for rural and remote regions in Canada where increased rurality leads to an increased risk for chronic disease and also limited access to cost-effective, evidence-based, chronic disease prevention programs and tools [16, 17].

The Health*e*Steps™ program was developed in response to the literature demonstrating the effectiveness of primary care-based chronic disease prevention programs [18], consultations with stakeholders and experts across Canada (including targeted discussions with knowledge users), and the need for such programs especially in rural and remote areas [19]. The Health*e*Steps™ program was developed from an extensive research base [18, 20–27]; draws on evidence from diverse areas including PA, nutrition, behaviour change, and knowledge translation; and has been refined by our experience implementing the program in diverse community settings including Family Health Teams, Community Health Centres, health clubs, and workplaces.

The Health*e*Steps™ program provides individuals with a specific plan of action to improve their PA levels, healthy eating habits, and reduce their sedentary behaviour. This is achieved through personalized coaching, grounded in the principles of Motivational Interviewing [28] and Co-Active coaching [29], and supported by innovative health services technologies, hands-on training, and widely available health promotion resources and tools. A distinct advantage of Health*e*Steps™ is the pragmatic nature of the program and ability to work within the existing workflow of an organization. While certain elements of the program must be conducted exactly as designed to follow the evidence-based protocols, other aspects are flexible. For example, the program may be conducted in-person in small groups or one-on-one; it can be run in a variety of primary care based settings; and it can be integrated with other existing programs.

## Methods

### Study aims

1. To conduct an outcome evaluation to determine effectiveness of the Health*e*Steps™ program in helping at-risk individuals increase PA levels (i.e., average number of steps per day), improve eating habits, and improve other health behaviours and health indicators.
2. To conduct a process evaluation to determine acceptability of the intervention, to inform program improvement and optimization.
3. To conduct an economic evaluation to determine the direct cost associated with delivering the Health*e*Steps™ program at different community sites, and the average cost per participant involved in the Health*e*Steps™ program.

### Study design

**Fig. 1:**
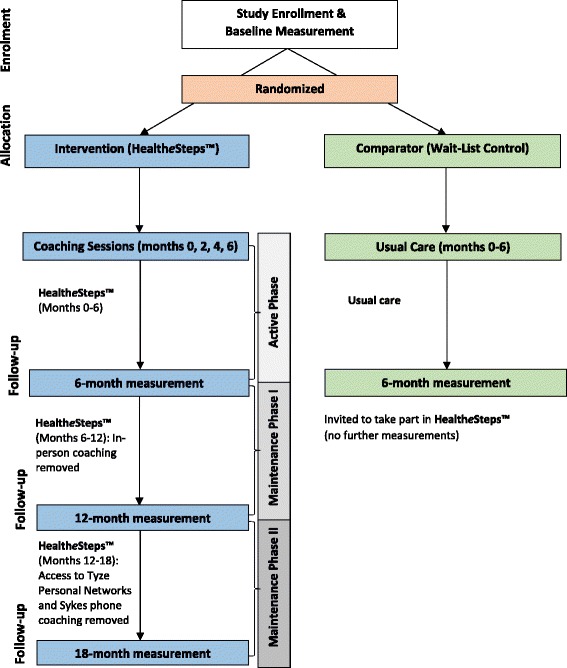
HealtheSteps Flow Diagram

A 6-month, two-arm, pilot, pragmatic, randomized controlled trial (RCT) with integrated process and economic evaluations will be conducted within five clinic/health care organization settings in both urban and rural communities in Southwestern Ontario. Participants will be individually randomized (1:1, stratified by site [three sites in London, one site in Forest, and one site in Tillsonburg, Ontario Canada]) to either the intervention (Health*e*Steps™) or the comparator (Wait-list control). Participants in both groups will complete measurement sessions at baseline and 6 months; participants in the intervention group will complete additional measurements at 12 and 18 months. Participants in the comparator group will be given the opportunity to start the Health*e*Steps™ program after a 6-month delay. See Fig. 1 for study flow diagram.

Study recruitment will begin in May 2015 at the 5 clinic sites, with the intervention starting in June 2015. Western University Health Sciences Research Ethics Board approved this study and all participants will provide written informed consent. The pragmatic RCT portion of this study was registered on April 6, 2015 with ClinicalTrials.gov (identifier: NCT02413385).

### Recruitment of study sites & coach training

This trial was designed to recruit primary care sites and/or health service organizations as they provide greater opportunities to recruit participants at risk or diagnosed with a chronic disease. We will solicit interest from primary care community sites for participation in the trial by contacting primary care leads in target communities where they had not been previously exposed to the Health*e*Steps™ program. Sites will be recruited opportunistically through connections with the research team. A variety of staff from each site will be recruited for training to be coaches in the program. Meetings with sites will occur with the central research team to ensure the coaches are trained in the program protocol and understand the commitment required (space, coaching staff, and timelines). Health*e*Steps™ coaches will be trained by the central research team or via *e*Learning modules available through the Health*e*Steps™ website [30]. In order to keep the trial highly pragmatic, coaching sessions and measurement sessions will occur at the sites.

### Sample size

We set a recruitment target of 110 participants. Baker and colleagues [31] conducted a 12-week RCT of a pedometer-based walking program plus PA consultation in 80 community-dwelling Scottish adults [mean (SD) age: 49.2 ± 8.8 years; 79% women] who were not meeting current PA recommendations (20% withdrawal rate by 12 weeks). After 12 weeks, this study reported a difference between intervention and control groups in mean change of 3021 steps per day – favouring the intervention group. Using a pooled standard deviation of 3498 steps per day results a Cohen’s d effect size of 0.86. In order to be conservative, we reduced the effect size to 0.65, a moderate to large effect size. Thus, with 38 participants per group, our study would have 80% power at a 0.05 two-sided significance level to detect an effect size of 0.65 [32]. We estimate a dropout rate of 30% over the 6-month period, which will increase our target to 110 participants (55 participants per group). Since we have 5 sites who expressed interest in taking part in this study, we propose that each site will need to enroll 22 participants (i.e., 11 participants per group per site).

### Participant recruitment & enrollment

Participants will be recruited at the sites through poster advertisements, word of mouth, health care provider referrals, staff e-mails, and in-person recruitment booths. Those interested in participating will be contacted by the central research team and asked to attend an in-person screening session. At this session they will be screened for eligibility and if deemed eligible will take part in baseline measurements. Randomization will occur after baseline assessments. All individuals who contact a study site will be asked to report where they heard about the program. Participants who are ineligible and those who choose not to enroll in the study after initial contact will be tracked, along with reasons for ineligibility/not enrolling. Acceptability of randomization will be estimated from the percentage of participants who adhered to their allocation assignment (i.e., attend program sessions if allocated to the intervention group) and/or attend measurement sessions. Participation in the follow-up measurement sessions will be used to assess retention throughout the trial. Attendance at program sessions will be obtained from coach’s logs and will be used to assess adherence to the program.

#### Inclusion criteria

Individuals 18–85 years of age; one or more self-reported or measured risk factors for chronic disease including: body-mass index (BMI) greater than 25 kg/m^2^; less than 150 min of exercise per week; 3 or more hours sitting per day; consuming less than 8 fruit and vegetable servings per day; diagnosis of metabolic syndrome or type 2 diabetes; and clearance to participate in PA via the Physical Activity Readiness-Questionnaire (PAR-Q) or a health care provider.

#### Exclusion criteria

Individuals who are unable to comprehend the letter of information and consent documentation.

### Randomization and allocation

Following the baseline measurement, participants at each site will be individually allocated to either the intervention (Health*e*Steps™ program) or comparator (Wait-list control). The randomization sequence (1:1, stratified by site) will be computer-generated and conducted by a member of the research team who will not be involved in allocation. Following allocation, all participants (intervention and comparator groups) will receive Eating Well with Canada’s Food Guide [33] and the Canadian Physical Activity Guidelines for Adults [34].

### Adverse events

Participants will be given information at baseline on reporting adverse events to the central research team throughout their participation in the study. Adverse events are described as any injury or newly diagnosed health condition (i.e., high blood pressure, diabetes) that occurs while a participant is enrolled in the Health*e*Steps™ study, whether or not it is related to their participation in the Health*e*Steps™ program. At each coaching session, participants in both the intervention and comparator groups will be asked about any adverse events they may have experienced while they were enrolled in the Health*e*Steps™ study, and whether it was related to their participation in the program. The adverse events will be reviewed by the lead study physician and if necessary, refer participants for appropriate event follow-up. The outcome of adverse events will be followed until the end of the study.

### Intervention group: Health*e*Steps™ Program

There are three phases to the intervention: an *active phase* that encompasses the first six months, a *maintenance phase I* for the next six months, and finally a *maintenance phase II,* which is the last six months of the trial.

#### Active phase (months 0–6)

Participants assigned to the intervention group will start the Health*e*Steps™ program within three weeks of completing their baseline measurement session. Participants will receive a handbook that contains documents to help them track their progress throughout the program and will be given a pedometer to use to track their PA. The research team chose to utilize pedometers to measure step counts as they are inexpensive, easy to use, and easily interpreted by the participant and research team [35]. In total, participants will receive four bi-monthly in-person coaching sessions (occurring at months 0, 2, 4, and 6) with a Health*e*Steps™ coach who will be trained on how to create an action plan with the participant to help them work towards achieving their lifestyle prescriptions. Strategies for setting lifestyle prescriptions are based on S.M.A.R.T. goal setting principles (specific, measurable, attainable, realistic, and timely for the participant), as goal setting has been found to produce positive behaviour change in adults [36]. The lifestyle prescriptions and supports are as follows:

1. *Exercise Prescription:* The exercise prescription focuses on making incremental changes in the amount of time the participant engages in moderate to vigorous activity or the amount of time they exercise within their target heart rate zone, up to the recommended 150 min/week. At each program session, participants will complete a sub-maximal fitness test, the Step Test and Exercise Prescription tool (STEP™ Test) [37]. Based on their fitness score from this test (i.e., predicted maximal oxygen uptake, VO2max), participants will receive an exercise prescription with a personalized target heart rate (65–85% of estimated maximum heart rate) for exercise. Coaches will then encourage participants to exercise at their training heart rate and/or at a rating of 5–8 on the 10-point modified Borg Rating of Perceived Exertion scale during aerobic activities [38]. The Health*e*Steps™ coach will collaborate with the participant to set a weekly target of exercise minutes that includes frequency, duration, and type of exercise to facilitate achievement of their exercise prescription.
2. *Physical Activity Prescription:* The PA prescription focuses on making incremental changes to the participants’ daily step count to reach the recommended 10,000 steps per day. This requires setting a baseline step count, which for coaching session 1 will be the participants’ step count recorded during the baseline measurement session. For sessions 2 to 4, the prescription will be based on steps logged in the participants’ handbook completed between sessions. If the participant did not track their step counts between coaching sessions, the coach and participant will work together to develop a reasonable target step count to set a new PA prescription.
3. *Healthy Eating Prescription:* Using Eating Well with Canada’s Food Guide, participants will work with their coach to determine their current daily intake from each food group, their water intake, and consumption of a healthy balanced breakfast. The healthy eating goal is set through increasing or decreasing a serving in one of the food groups to better meet the recommendations (i.e., increasing one serving of vegetables and fruit or decreasing a serving of meats and alternatives), increasing water intake and increasing the number of days the participant consumes a healthy balanced breakfast.
4. *Access to the HealtheSteps™ eHealth Technology Suite:* Tyze Personal Networks [39] and Sykes [40] phone coaching will provide participants with support in between coaching sessions and will be available during the *maintenance phase I* until the 12 month assessments are complete. Tyze Personal Networks is a web-based platform that provides an exclusive network for connecting with central research staff, coaches, and other participants through private chat services and posting information on a CareWall (similar to a Facebook wall). Sykes provides phone coaching services to support participants in-between coaching sessions and during *maintenance phase I*. Sykes CareCoaches® are trained in the Health*e*Steps™ protocol and act similarly to the Health*e*Steps™ coach, working with participants to address any challenges they may be experiencing and providing encouragement for them to meet their goals.
  Participants will be encouraged to seek out publicly available healthy living supports, such as the Health*e*Steps™ website (http://healthesteps.ca/) for healthy living resources and the Health*e*Steps™ and eaTracker smartphone apps. The Health*e*Steps™ app (available for free download on Apple and Android devices) has a built-in fitness test and heart rate monitor. Additionally, through the help of a virtual coach, the app provides personalized prescriptions, goals, and tracking options to help participants maintain their goals developed with their in-person coach. The eaTracker allows participants to monitor food and activity choices, analyze recipes, and create meal plans. These publicly available supports are available to participants throughout the active and maintenance phases as well as beyond the trial period.

Between coaching sessions, participants will be expected to self-direct their healthy living activities, using healthy living resources (Eating Well with Canada’s Food Guide and Canadian Physical Activity Guidelines) and *e*Health technology support options.

#### Maintenance phase I (months 6–12)

Participants will no longer have in-person coaching sessions, but will have access to the full suite of *e*Health technology support tools.

#### Maintenance phase II (months 12–18)

Participants will no longer have access to the online Tyze Personal Networks, and Sykes phone coaching services, but will still have access to the publicly available resources, including the HealtheSteps™ and eaTracker Smartphone apps, and the Health*e*Steps™ website.

### Comparator group (wait-list control)

This group will continue with usual activities without intervention from the study team for the first 6-month period. After the 6 month measurement sessions are completed, participants allocated to this group will be given the opportunity to start the 6-month Health*e*Steps™ program.

### Data collection

#### Outcome evaluation

The outcome measures will be taken at baseline and 6 months in both groups, and then again at 12 and 18 months in the intervention group only (see Table 1). Home visits will be conducted when participants are unable to attend follow-up measurement sessions at the sites to increase participant retention throughout the long-term follow-up. Data sources will include self-reported PA, participant demographics, clinical measures, and the completion of health related information and questionnaires (See Table 2).

**Table 1:** Screening and measurement sessions schedule

Measurement	Screening	Baseline	6-month	12-month (Intervention Group only)	18-month (Intervention Group only)
*Screening & Eligibility Measures*					
Participant Self-Reports at Screening	X				
Pre-Randomization ID #	X				
Letter of Information & Consent Form	X			X	
PAR-Q	X				
Health Care Provider Clearance Form	X				
Eligibility Form	X				
Group Allocation		X^a^			
Participant ID#		X^a^			
*Baseline Health Information*					
Demographics & Health Related Information		X			
*Clinical Measures*					
Age	X	X^b^			
Height	X	X^b^	X^b^	X^b^	X^b^
Weight	X	X^b^	X	X	X
Body Mass Index	X	X^b^	X	X	X
Waist Circumference		X	X	X	X
Blood Pressure		X	X	X	X
*Self-Reported Physical Activity*					
Average Steps/day (Step Count Outcome Tracking Form)		X	X	X	X
International Physical Activity Questionnaire		X	X	X	X
*Self-Reported Eating*					
Modified DINE		X	X	X	X
Starting the Conversation		X	X	X	X
*Health-Related Quality of Life*					
EQ-5D-3 L		X	X	X	X

^a^Occurs at Randomization & Allocation Session

^b^Use number recorded at Screening Session

**Table 2:** Data collection & measurement protocol

Outcome Measure	Equipment required	Protocol
*Self-Reported Physical Activity*		
Average steps/day	Pedometer (Yamax Digiwalker SW-200 with security strap)	• Participants wear pedometers during waking hours for a 7-day period (putting pedometer on upon waking and removing immediately before sleeping), but not during showering/bathing • Participants are asked to wear the pedometer at the waist, centered over their most dominant foot • Participants record the number of steps completed each day on a paper-based tracking form
Total physical activity (MET-min/week^a^)	International Physical Activity Questionnaire (IPAQ) – Short Form [43]	• Self-completed paper‐based questionnaire • Participants recall information on vigorous activities, moderate activities, walking, and sitting • Sedentary time is measured with a single question (minutes spent sitting on a typical week day) • The IPAQ provides guidelines for data processing and score creation
Time spent in sedentary activity (min/day)		
*Clinical Measurements*		
Weight (kg and percentage of baseline weight)	Digital Weight Scale (Tanita HD 351)	• Light clothing, no shoes and empty pockets • Blinded assessor post-baseline in private area
Body mass index (kg/m2)	Digital weight scale (Tanita HD‐351) Portable stadiometer (seca 213)	• BMI calculated using the participant’s objectively measured height and weight • Height measured without shoes
Waist circumference (cm)	Tape measure	• Follows protocol outlined by the Heart and Stroke Foundation of Canada [44] • Two measurements taken to record an average; if measurements differ by more than 5 mm, a third measurement is taken and used in the average
Systolic Blood Pressure (mmHg)	Digital BP monitor (BP Tru BPM-100)	• Participants sit quietly for 5 min prior to the first measurement; 3 measurements will be taken, 2 min apart. The first one is discarded, and the average of the last two is recorded. • Feet flat on the floor, arm free of clothing, cuff at the level of heart and arm resting, same arm used (left arm preferred), no talking
Diastolic Blood Pressure (mmHg)		
*Self-Reported Eating*		
Fatty Food Score	Modified version of the Dietary Instrument for Nutrition Education (DINE) [46]	• Self-completed paper-based questionnaire • Participants recall eating habits over the last 7 days • Methods published by FFIT will be followed to calculate a fatty food score (possible range 8–68) and sugary food score (possible range 3–16), with higher scores indicative of higher consumption • Fruit and vegetable consumption is measured with a single question
Sugary Food Score		
Fruit and vegetable consumption		
Total healthful eating score	Starting the Conversation [45]	• Self-completed paper-based questionnaire • Designed for dietary assessment and intervention in a clinical setting • Participants recall eating habits over the past few months (on average) on 8 different items • A total healthful eating score (possible range 0–16) is calculated, whereby a lower score indicates a more healthful diet
*Health-Related Quality of Life*		
Self-Rated Health [visual analog scale (VAS) score]	European Quality of Life 5 Dimensions Questionnaire - 3 Level Version (EQ‐5D- 3 L) [48]	• Self-completed paper-based questionnaire • For purposes of this study, the VAS score will be used to assess current state of health on a scale from 0 (worst imaginable state of health) to 100 (best imaginable state of health)

^a^METs (metabolic equivalents) are multiples of the resting metabolic rate; MET-minute = multiplying the MET score of an activity by the minutes performed

1. Average steps per day, measured using Yamax Digiwalker SW-200 pedometers and self-reported by participants using a 7-day paper log [41, 42] (at least 3 days of logging required).
2. Total physical activity (Metabolic Equivalent (MET)-minutes/week) measured using the International Physical Activity Questionnaire (IPAQ) Short Form [43]; and time spent in sedentary activity (minutes spent sitting on a typical week day) measured with the IPAQ.
3. Objectively-measured clinical characteristics: weight (using Tanita HD 351 Digital Weight Scale; kg and % of baseline weight), Body Mass Index (BMI, calculated from weight and height in kg/m^2^), waist circumference (cm) [44], and resting systolic blood pressure (BP) and diastolic BP (using BP Tru BPM-100; mmHg).
4. Self-reported eating: healthful eating score, measured using Starting the Conversation (STC) questionnaire [45]; fatty food score, sugary food score, as well as fruit and vegetable consumption, measured using a modified version of the Dietary Instrument for Nutrition Education (DINE) [46] and following scoring outlined by Hunt and colleagues [47].
5. Self-rated health measured using the European Quality of Life – 5 Dimensions – 3 Levels (EQ-5D-3 L) visual analog scale (VAS) score [48, 49].

#### Process evaluation

Data collection for the process evaluation will also be collected during baseline, 6, 12, and 18 month measurement sessions (see Table 3). Information about how participants heard about the program, demographic information, interviews with coaches, participants and program non-completers, questionnaires completed by participants, and participant compliance and retention will inform the acceptability of the intervention.

**Table 3:** Data sources to address process measures

Process measures	Data sources
Program Reach	Participant Screening and Baseline Demographics
	12-Month Participant Program Questionnaire
	12-Month Participant Interview
Reasons Participants Stayed With/Opted Out of the Program	Interviewer-Guided Participant Feedback Questionnaires
	Program Compliance Records
	Non-Completer Telephone Interviews
	12-Month Participant Program Questionnaire
	12-Month Participant Interview
Extent to which Coaches Delivered Health*e*Steps as Designed	Coach Interviews
Participants’ Experience Taking Part in Health*e*Steps	Interviewer-Guided Participant Feedback Questionnaires
	12-Month Participant Program Questionnaire
	12-Month Participant Interview
	18-Month Participant Program Questionnaire
Coaches’ Experience of Delivering Health*e*Steps	Coach Interviews
Participants’ Experience Maintaining Lifestyle Changes	12-Month Participant Program Questionnaire
	12-Month Participant Interview
	18-Month Participant Program Questionnaire

**At screening and baseline**, participants will be asked how they heard about the study and demographic information will be taken.

**At 6 months**, participants who completed the Health*e*Steps™ program will be asked to complete an Interviewer-Guided Program Feedback Questionnaire; in order to capture all relevant information about the program, participants will be guided through the feedback forms by a trained interviewer, in-person or by phone. Participants will be asked about how useful they found different program components, benefits they may have experienced through the program, helpful methods for keeping on track with their healthy lifestyle goals, barriers and challenges in maintaining their goals, and experience using the *e*Health technology suite.

Health*e*Steps™ coaches will have the opportunity to express their thoughts on delivering the Health*e*Steps™ program through an interview conducted by a trained interviewer in-person or through the phone. Interviews will be kept anonymous with questions exploring their experience delivering the Health*e*Steps™ program to participants, preparedness for program delivery, effectiveness of different program components and suggestions to improve the different program components. Prior to these interviews, coaches will be asked to read through a letter of information and sign a consent form allowing the interviewer to audio record the interview for more thorough analysis.

Participants who are enrolled in the study but do not complete any coaching sessions or complete only one coaching session, half of the coaching sessions or do not attend sessions 3 or 4, will be defined as non-completers. Program non-completers will be asked in-person or via phone, a series of questions about why they joined the Health*e*Steps™ program, barriers for participating in the program, and changes they may have made during their enrollment in the program.

All data sources at 6 months will be kept anonymous in order to encourage open and honest answers and prevent bias during the data analysis stage.

**At 12 months**, participants will be asked to complete a 12-month Program Questionnaire detailing what they have been able to maintain throughout *maintenance period I,* and whether they used the *e*Health technology support tools. Participants will also be asked to participate in an interview exploring their experience maintaining their health behaviour changes 6 months after the *active phase*. Questions will explore their overall experience with the program; their experience maintaining the changes in exercise, PA levels, and eating habits; the *e*Health technology support tools; and impact their participation in the program may have had on friends and family.

**At 18 months**, participants will be asked to complete a final questionnaire about their experience maintaining healthy lifestyle changes one year after completion of the active phase. Questions will also focus on support systems they may have used to maintain their lifestyle changes as Tyze Personal Networks and Sykes Phone Coaching supports ended at 12 months.

### Data analyses

#### Outcome evaluation – statistical (quantitative) analysis

The primary outcome will be the difference between the intervention and comparator groups in mean steps per day at 6 months. For all secondary outcomes, we will also examine differences between intervention and comparator groups in mean change at 6 months. The additional follow-up data collected from Health*e*Steps™ group will allow us to evaluate: i) feasibility of retaining individuals for an extra 6 and 12 months (to inform a larger study); and ii) within group change to 12 and 18 months to determine the sustainability of any positive changes (in health behaviours and health indicators) observed following the active phase.

We will analyze data based on an intent-to-treat approach; thus, we will include all participants with at least valid baseline data according to the randomization scheme. We will analyze data using linear or generalized linear mixed models for repeated measurements and we will retain the baseline outcome as part of the outcome vector and constrain the group means as equal because of randomization [50]. This approach is equivalent to analysis of covariance approach, but has the advantage of including subjects with missing data [51]. For all models, we will examine differences between groups at 6 months and changes within groups from baseline to 6 months (and to 12 months and 18 months for the intervention group). Terms included in models will include time, treatment (group) × time, age and site. Time will be modeled categorically with indicator variables (with baseline as the reference category). Residuals from models will be examined and subject to assumptions checks.

To address participant dropout, we will also compare baseline characteristics of participants who dropped out versus participants who were included in the analysis. Interpretation of study results will primarily be based on estimation and associated 95% confidence intervals [50]. Confidence intervals for differences excluding zero or two-sided p-values less than 0.05 will be reported as statistically significant. Analyses will be performed using SAS version 9.4 (SAS Statistical Analysis Software).

#### Process evaluation – qualitative analysis

Qualitative data sources will include the Interviewer-Guided Participant Feedback Questionnaires and coach interviews collected at the 6 month assessments and the participant interviews collected at the 12 month assessments. Due to the high volume of participant interviews at 12 months, a representative sample (maximum variation based on participant baseline demographics and clinical measures) of interviews from each site will be selected for transcription and analysis; these data sources will be analyzed separately by members of the research team whom will not be involved in direct program delivery. An inductive content analysis will be performed. The research team will read through all of the transcripts and identify common themes and exemplar quotes that represent this theme; following a group discussion, a final list of overarching themes will be created. The findings from the Interviewer-Guided Participant Feedback Questionnaires, and coach and participant interviews will be triangulated to produce a detailed description of the participants’ experience with the Health*e*Steps™ program, the coaches’ experience delivering the program, and the participants’ experience maintaining their changes. This will inform future optimization of the Health*e*Steps™ program.

#### Economic evaluation

The economic evaluation will be conducted by the Centre for exceLlence in Economic Analysis Research (CLEAR) Team. The primary analysis will consist of calculating the total cost of the Health*e*Steps™ program. Total costs will be calculated by summing all item costs, regardless of site, study group, payer perspective, component, and time period. Total cost will then be broken down for each study group and site. Secondary analysis involves calculating the average cost per person of the Health*e*Steps™ program. The average cost per person will be calculated by dividing the total cost of the Health*e*Steps™ program by the number of participants at each site. Given that participants differ by site, per person cost will be presented by site and by study group (intervention vs. comparator). Accordingly, total cost of the program will be broken down for each study group and site, and subsequently by component, payer perspective, and time period. The same process will be used to calculate the average cost of the Health*e*Steps™ program per participant. The final analysis stage will estimate the total and average per person cost to implement the Health*e*Steps™ program at a new site. Total costs will be calculated by summing all item costs that contributed to the implementation of the Health*e*Steps™ program at a new site. The average cost per person will be calculated by dividing the total cost of the Health*e*Steps™ program by the number of assumed participants and coaches (a participant to coach ratio of 15:1) at this hypothetical site. Both the total and average per person cost will also be categorized by component costs (i.e., personnel, supplies and miscellaneous, and supports and technology).

## Discussion

Living a physically active lifestyle along with consuming a healthier diet and reducing tobacco intake can prevent 80% of chronic diseases, such as heart disease, stroke, and type 2 diabetes, and 40% of cancer cases [10]. The majority of Canadian adults continue to spend a significant amount of their waking hours being physically inactive and only 39.5% indicate that they consume 5 or more fruits and vegetables per day [3, 52]. The general population is not aware of the PA and exercise guidelines [53], and how to use Eating Healthy with Canada’s Food Guide to make healthy eating choices [54]. Therefore, interventions that promote healthy lifestyles need to include educational resources and guidance to ensure people are aware of these guidelines and how to use this knowledge to reduce their risk for chronic disease.

Health*e*Steps™ is a lifestyle program developed from an extensive research base and designed to provide participants with action specific goals and strategies to increase their PA and exercise levels, and encourage healthy eating habits, within the confines of their primary health care space. Health*e*Steps™ engages participants through multiple avenues, including using technology and phone supports to provide participants with the tools needed to make and maintain a healthy lifestyle. In this study we aim to determine the effectiveness of the Health*e*Steps™ program in helping at-risk individuals increase PA and exercise levels, and improve eating habits. This study also aims to determine the acceptability of the program to inform future optimization. Lastly, this study aims to determine the direct cost per participant involved in the program, and the cost to the community or site running the program. If the results are promising, our next step will be to further scale-up the program into rural and remote communities focusing on delivery in primary care, workplace wellness, and community-based facilities.

## Abbreviations

BMI: Body Mass Index
BP: Blood pressure
DINE: Dietary Instrument for Nutrition Education
EQ-5D-3 L: European Quality of Life – 5 Dimensions – 3 Level
IPAQ: International Physical Activity Questionnaire
MET: Metabolic Equivalent
PA: Physical activity
PAR-Q: Physical Activity Readiness Questionnaire
pRCT: Pragmatic Randomized Controlled Trial
STC: Starting the Conversation
STEP: Step Test and Exercise Prescription
VAS: Visual analog scale.
